# The latitudinal dependence in the trend of snow event to precipitation event ratio

**DOI:** 10.1038/s41598-021-97451-9

**Published:** 2021-09-13

**Authors:** Shangyong Shi, Guosheng Liu

**Affiliations:** Department of Earth, Ocean and Atmospheric Science, Florida State University, Tallahassee, FL USA

**Keywords:** Hydrology, Hydrology

## Abstract

Precipitation phase is expected to shift from solid to liquid with temperature rising, which would in turn bring challenges to regional water resource management. Although in recent decades, consistent decreasing trends in the ratio of snowfall to precipitation rate in a warming climate have been found across multiple regions, a global view of the trends in the precipitation partitioning has not been established. In this study, we investigated the global trends of annual rain and snow frequency of occurrences and the ratio of number of snow events to number of precipitation events (SE/PE ratio) using land station and shipboard synoptic present weather reports from 1978 to 2019. Results show that when averaged over all qualified land stations and over the shipboard reports, both the annual rain frequency and snow frequency decrease over the 42 years. Over both land and ocean, the averaged SE/PE ratio has a significant decreasing trend. Moreover, the trend of SE/PE ratio shows a strong latitudinal dependence. At the mid- and low latitudes in the Northern Hemisphere, the SE/PE ratio has a decreasing trend. In contrast, at high latitudes, the SE/PE ratio has an increasing trend.

## Introduction

Global warming is reported to induce changes in precipitation^1,2^. Among all the precipitation characteristics, precipitation phase, i.e., solid or liquid, significantly influences snow-driven hydrology. At high elevations and high latitudes, since surface temperature there often stays below freezing point, increases in precipitation amount have resulted in increased snowpack, while at mid and low elevations and latitudes, warmer winter temperature reduces snowpack as more precipitation falls as rain instead of snow^3–5^. The ratio of snowfall amount to total precipitation amount (S/P ratio) is often regarded as an indicator for precipitation phase partitioning. A lower S/P ratio and earlier snowmelt^6,7^ would result in higher winter and spring runoff rates, larger risk of winter and spring floods, and lower summer and fall water supply^8–11^. In addition, the S/P ratio would affect surface albedo through changing the area of snow-covered surface, thus alter energy budget at the surface and influence global weather and climate^12^.

Decreasing trends of S/P ratio in the later part of twentieth century have been found across different regions^12–18^. In these studies, the decreases in snowfall amount were consistent, but the trends in total precipitation vary among regions. The ratio of snowfall days to precipitation days was also found to decrease over the past century in Switzerland^19^, with the decreasing trend stronger at lower elevations where temperatures were closer to the melting point. Such correlation of S/P ratio with temperature was confirmed in multiple studies^14,19,20^, and significant correlations with large scale climate patterns like the North Atlantic Oscillation and the Pacific-North American indices were also observed^16,17^. Most of the studies relied on snow water equivalent determined from gauge-measured precipitation amount, which is often biased due to environmental factors and equipment issues^21,22^, especially over windy high latitudes^22–24^. Additionally, due to scarce distribution of gauges and differences in catch efficiency between the national standard gauges^22^, the evaluation of S/P ratio were usually limited to a certain country or local region, leaving the global pattern of the precipitation phase partitioning unknown.

Present weather code in synoptic reports from land manual weather stations and ships can be used to fill the gap in studying precipitation events on the global scale. Defined by the World Meteorological Organization (WMO), it ranges from 0 to 99 and is referred to as “ww” code^25^. It provides valuable information on station weather conditions including precipitation phase and type, making it possible to evaluate spatial distributions or time evolutions of different weather events^26,27^.

In this study, we utilized the present weather code to separate rain and snow events, and then analyzed the trends in frequency of occurrences of rain and snow events, and the ratio of number of snow events to number of total precipitation events (SE/PE ratio) from 1978 to 2019. Snow events are defined to include only solid precipitation with “ww” code from 70 to 79, 85 and 86; rain events include all liquid and mixed phase precipitation with “ww” code from 60 to 99 excluding those for snow^28^. Detailed data and methodologies are described in the “Methods” section, and “Section 1: Merging data sets” and “Section 2: Data cleaning” in “Supplementary Information”. Data from 3915 land stations and 713 ocean grids (5° × 5°) were analyzed (see Supplementary Fig. S2, “Supplementary Information”). Because of the broad coverage of land surface stations and ships, this study is able to assess the trend of the SE/PE ratio over a global scale (at least in the Northern Hemisphere), which fills the gaps left by the aforementioned regional studies, therefore, improves our understanding on the changes in the fraction of precipitation falling as snow rather than rain in a warming climate. Considering the difference in data density and quality between land station and ocean ship reports, readers are cautioned to interpret land and ocean results differently.

## Results

### Overall trends

**Figure 1:**
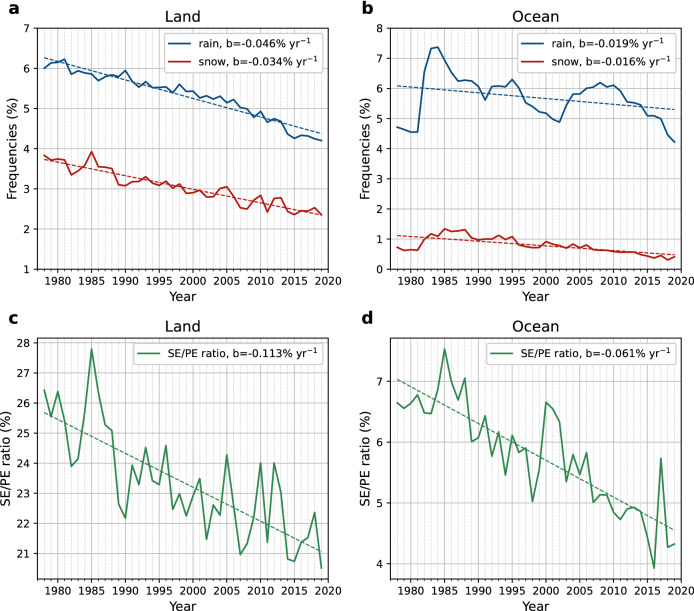
Time evolution of frequencies of rain and snow events. (**a**, **b**) Frequency of occurrences of rain and snow events averaged over qualified land stations (**a**) or ocean grids (**b**). (**c**, **d**) Ratio of snow events to precipitation events (SE/PE ratio) averaged over qualified land stations (**c**) or ocean grids (**d**). Linear least squares fitting to the data is applied with slopes shown in the legends as "b" (unit: fraction year^−1^).

When averaged over the qualified land stations, significant decreasing trends are found both in the rain and snow frequency for the 42 years (Fig. 1a). The snow frequency decreases at a rate of 0.034% year^−1^, while the rain frequency decreases faster at a rate of 0.046% year^−1^. Over ocean, as shown in Fig. 1b, the rain frequency has a magnitude similar to that over land but decreases at a smaller rate (0.019% year^−1^). The snow frequency over ocean also decreases during the 42 years (− 0.016% year^−1^). Note that the rain and snow frequency over ocean suddenly increased around 1982. This was the year when WMO applied a new coding practice, which led to changes in the number of oceanic ww reports^26,29^. Though we plotted the trend for ocean from 1978 to 2019, more credit should be given to the period starting from around 1983, when the practice became fully adopted. As a reference, the trends from 1983 to 2019 for the rain and snow frequency are − 0.049% year^−1^ and − 0.033% year^−1^, respectively.

The averaged SE/PE ratios over land and ocean both show a significant decreasing trend (Fig. 1c,d). The value of SE/PE ratio over ocean is almost a quarter of that over land, which may be partially because ships tend to avoid sailing on snowy days. Due to this possible sampling issue, in the following sections we would only present the calculation results without comparing ocean with land. The SE/PE ratio over land decreases from around 26 to 21%, at a rate of − 0.113% year^−1^, while over ocean it reduces from 7 to 4.5%, at a rate of − 0.061% year^−1^, which is about half as that of land. The trend from 1983 to 2019 for ocean is − 0.11% year^−1^ for the readers’ reference.

### Global distribution of means and trends of precipitation frequency

**Figure 2:**
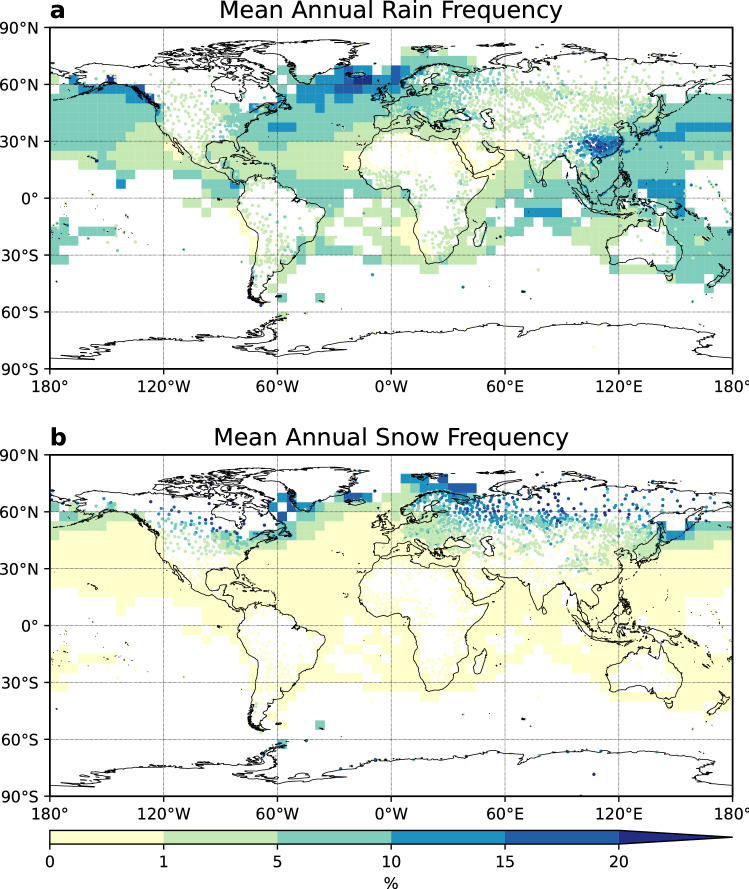
Mean annual rain and snow frequency of occurrences. (**a**) Mean annual rain frequency. (**b**) Mean annual snow frequency. Rain (snow) frequency of occurrence was calculated for each year, then the 42-year mean were computed for 1978–2019. This figure was plotted using Python 3.7.4 (https://www.python.org/downloads/release/python-374/).

The annual means of rain and snow frequency were calculated for each qualified land station and ocean grid (Fig. 2). Regions with rain frequency smaller than 5% include most parts of the North and South America, Africa, the Middle East, Central Asia, Siberia, northern China, Australia and some parts of the sub-tropical oceans. China has dense and continuous observations and features large annual rain frequency of more than 10% in southern China. Other stations with > 10% rain frequency locate in East Canada, Iceland, United Kingdom, Norway, Alpine mountains, around the Black Sea, Southeast Asia, the islands east of Australia, as well as in a few grids in the West and North Pacific Ocean, North Atlantic Ocean and Central Indian Ocean. Rain frequency of more than 20% appear in the coastal region of Gulf of Alaska, the southern tip of South America, Southwest China and the ocean south of Iceland.

Over land in the Northern Hemisphere, snow starts to appear around 35° N. The snow frequency increases with latitude, with the largest frequency of more than 20% occurring mostly north of 50° N (Fig. 2b). In the Southern Hemisphere, snow is observed in the southern tip of the South America and stations in the Antarctica. Over ocean, the snow frequency is mostly below 1%. However, previous studies using CloudSat data showed that snowfall frequency can be higher than 10% in some regions^30,31^, which suggests that the snow frequency derived from ship weather reports here is biased low. Values larger than 1% over ocean locate in the northwestern and northeastern coastal area of the Pacific Ocean and Atlantic Ocean, and in a few grids in the Antarctic Ocean.

**Figure 3:**
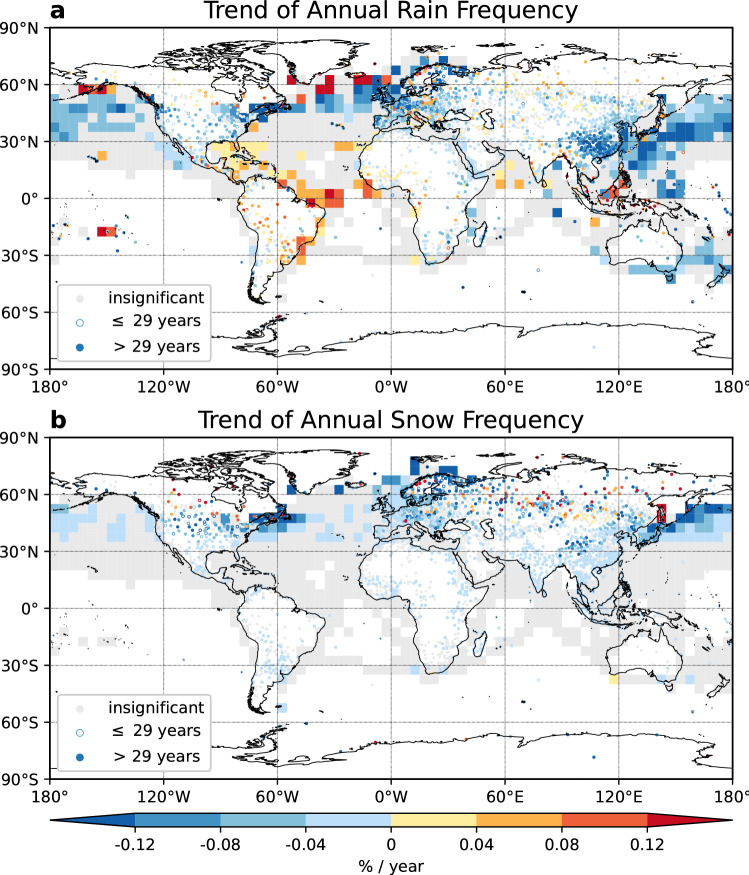
Trends of annual rain and snow frequency of occurrences. (**a**) Trends of annual rain frequency (in % year^−1^). (**b**) Trends of annual snow frequency for 1978–2019 (in % year^−1^). Insignificant trends are shown in gray. Stations with data length between 25 and 29 years are marked as open circles, and those with data length longer than 29 years are marked in filled circles. Stations with less than 25-year qualified data and grids with less than 30-year qualified data for calculating trends are masked out. This figure was plotted using Python 3.7.4 (https://www.python.org/downloads/release/python-374/).

The trends of rain and snow frequencies for each qualified land station and ocean grid are shown in Fig. 3. Land stations with data length between 25 and 29 years were marked as open circles, while stations with more than 29-year data were plotted in filled circles. Over ocean, trends were calculated for grids with more than 30-year data. For the annual rain frequency, decreasing trends dominate most continental regions (Fig. 3a), including the Central North America, Europe, Central Asia, East Asia and Australia. Over land, significant decreasing trends stronger than − 0.12% year^−1^ are observed in Alaska, western and northern Europe, eastern coast of the Baltic Sea, southwestern China, Japan and islands in the Indian Ocean and the Pacific Ocean. Over ocean, the West and North Pacific Ocean, North Atlantic and East Indian Ocean also feature decreasing trends. Increasing trends of rain frequency over land are mostly found in tropical stations and ocean grids, i.e., Central America, South America, South Asia and Southeast Asia. Outside the tropics, some high latitude stations in Canada, central Europe, Norway and Russia and ocean grids in North Pacific and North Atlantic Ocean also have increasing trends of rain frequency.

The significant trends of annual snow frequency appear mostly in the Northern Hemisphere north of 30° N, corresponding to the distribution of annual mean snow frequency in Fig. 2b. Land stations are dominated with significant negative trends. The magnitude of negative trends increases with latitude. Positive trends of snow frequency locate between 50° N and 65° N. Over ocean, decreasing trends appear near eastern coast of Canada, North Atlantic Ocean, north of Scandinavian Peninsula and near the coastal regions in the Northwest Pacific Ocean. In the Southern Hemisphere, only some stations in the Antarctica have significant trends. Insignificant trends are almost everywhere over ocean, possibly due to ships' tendency to avoid snow events.

### Global means and trends of snow event to precipitation event ratio

**Figure 4:**
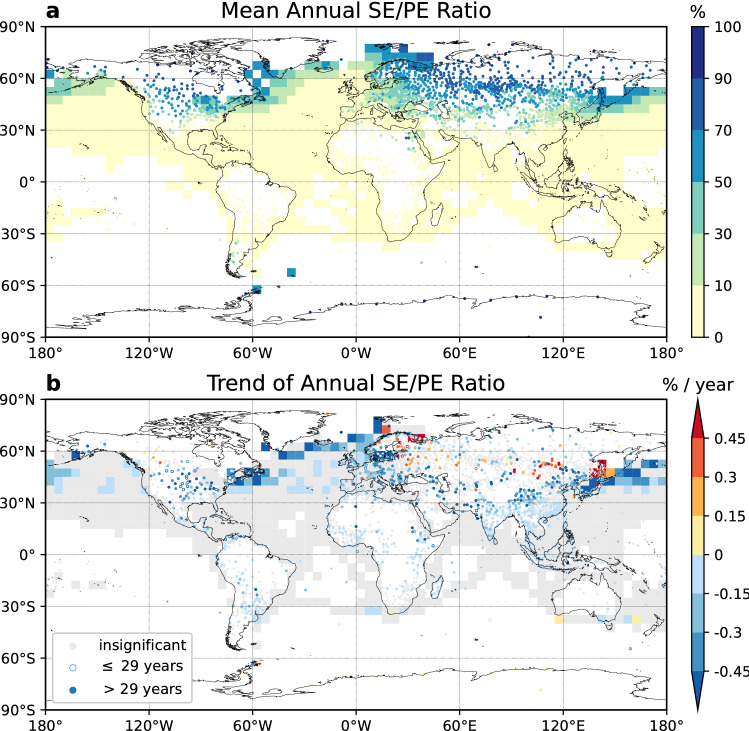
Means and trends of annual snow event to precipitation event ratio. (**a**) Global spatial distribution of mean annual snow event to precipitation event ratio (SE/PE ratio) for 1978–2019. (**b**) Trends (in % year^−1^) of annual SE/PE ratio between 1978 and 2019. Trends were not calculated for the tropical oceans (30° S–30° N). Insignificant trends are shown in gray. Stations with data length between 25 and 29 years are marked as open circles, and those with data length longer than 29 years are marked in filled circles. Stations with less than 25-year qualified data and grids with less than 30-year qualified data for calculating trends are masked out. This figure was plotted using Python 3.7.4 (https://www.python.org/downloads/release/python-374/).

The annual SE/PE ratio (Fig. 4a) indicates the possibility of a precipitation event to be snow. For the continents in the Northern Hemisphere, the SE/PE ratio exceeds 10% at around 30° N and grows larger as latitude gets higher. It reaches 70% at around 50° N in North America and Eurasia, and peaks at around 70° N. The value is below 10% for most ocean regions and exceeds 30% near the coastal areas in the Northwest Pacific Ocean, Northwest Atlantic Ocean and the Barents Sea north of Scandinavian Peninsula. As mentioned earlier, the SE/PE ratio over ocean may be underestimated due to possible under-sampling of snow events by ships.

The significant trends of annual SE/PE ratio are found mostly in the Northern Hemisphere, where the data are more abundant than those in the Southern Hemisphere (Fig. 4b).

In the tropics, many land stations have significant trends in the SE/PE ratio though the annual mean SE/PE ratio is below 1%. This is because these stations experienced several snow events in the early 1980s or 1990s but are capturing fewer and fewer snow events in recent years (see Supplementary Fig. S3 for an example). Over the tropical oceanic regions between 30° S and 30° N, the weather is relatively warm and there are very few snow events recorded. Since the large and significant increasing trend in the rain frequency in these regions would lead to the decreasing trend in the SE/PE ratio which is not realistic, we did not calculate the trends of the SE/PE ratio in these tropical oceanic regions.

In the Northern Hemisphere outside the tropics, decreasing trends of the SE/PE ratio over land occur mostly south of 45° N, with values lower than − 0.45% year^−1^ appearing in the central U.S, Europe, West Asia, South and East Asia. The decrease of SE/PE ratio in Canada, western and central US, UK and Tibetan Plateau is consistent with previous studies on S/P ratio^13,15,16,18^.

Increasing trends of annual SE/PE ratio are observed in higher latitudes. Positive trends mostly larger than 0.15% year^−1^ are observed in a few stations in western Canada, southern and northern Europe, the European Russia, stations east of Lake Baikal, and east Siberia coast. Most of these regions are poleward of 45° N, relatively cold and snow dominated areas (Fig. 4). Similarly, for the land stations in the Southern Hemisphere, increasing trends are observed poleward of 50° S.

## Conclusions and discussions

The number of rain and snow events at land stations and ships over ocean from 1978 to 2019 are examined based on synoptic present weather reports. Major findings include.

1. When averaged over the selected land stations or ocean grids, both rain and snow frequencies decrease during 1978–2019. The SE/PE ratio decreases at rate of − 0.113% per year over land and − 0.060% per year over ocean.
2. Decreasing trends of rain and snow frequency are observed in most regions. Increasing trends of rain frequency are mostly observed in the tropics. Large decreasing trends of rain frequency appear in coastal area of the North America, Europe and East Asia. Negative trends of snow frequency grow in magnitude with latitudes, with maxima occurring at high latitudes (around 60° N).
3. A latitudinal dependence of global pattern of the trends in the snow event to precipitation event ratio is observed. Increasing trends of SE/PE ratio are observed in high latitudes, while decreasing trends are observed in mid- and low latitudes.

In this study, we observed a latitudinal pattern of the trends in the SE/PE ratio which had not been reported before. The increasing trends in the SE/PE ratio in higher latitudes indicate that snow events are taking a growing portion in precipitation events in recent decades. The decreasing trends in the SE/PE ratio correspond to previous studies about the S/P ratio, but note that the SE/PE ratio defined in our study is different from the S/P ratio that focused on snowfall and precipitation amount. The SE/PE ratio emphasized "frequency of occurrences" of rain or snow events. Fewer portions of snow events do not necessarily mean less snowfall amount considering some of the events may be more extreme. How the distribution of snowfall rate or rainfall rate changes is out of our scope. Readers should keep this in mind when we discuss the patterns of the trend of SE/PE ratio and possible mechanism lying behind the changes.

Statistically, the trends in the S/P ratio were attributed to the relative changes in snow amount (dS/S) versus that in precipitation amount (dP/P)^32^. Similarly, if the relative increase (decrease) in the number of snow events exceeds that in the number of precipitation events, the station would exhibit an increasing (decreasing) trend in the SE/PE ratio. When we examined the time series station by station, we observed different combinations of the relative changes in snow and precipitation events when there is a significant trend in the SE/PE ratio. The number of snow and rain events can be (1) increasing or decreasing simultaneously, but one of them evolving faster than the other, (2) only one of them having a significant trend, or (3) one increasing and the other decreasing. Two typical examples are given in Supplementary Fig. S4, “Supplementary Information”.

From a physical viewpoint, the precipitation phase could be influenced by a number of meteorological variables including temperature, moisture, pressure, atmospheric vertical structure (e.g., lapse rate, freezing level height), etc.^28,33–36^. For example, high temperature is associated with small S/P ratio and vice versa. A high amount of moisture in the air at a given temperature would increase the melting rate of snowflakes, thus decrease the probability of snow event^34^. Comparing with total precipitation variability, temperature contributes more to determining the S/P ratio^14,19,37,38^, and the increase in temperature can result in either decrease or increase in S/P ratio at different stations depending on the magnitude of the changes induced in S and P^32^. In our study, the latitudinal dependence of the trends in SE/PE ratio is also related to temperature regimes. That is, among total precipitation events, colder areas are experiencing an increasing trend of the portion of snow events and warmer areas are experiencing an increasing of the portion of rain events (Fig. 4b). Our calculation indicates that the correlation coefficients between SE/PE ratio and surface temperature for 1978–2019 are stronger than − 0.6 over most of the land stations with significant trends (Supplementary Fig. 5, “Supplementary Information”). Analogous to the impact of increasing precipitation on snowpack^3–5^, the reason why we have increasing trends in the SE/PE ratio in high latitudes may be that the increased precipitation falls in the form of snow when the temperature is far below the freezing point. However, in this study, we did not include detailed relationships between temperature changes and the trends in SE/PE ratio. This can be explored in future studies.

Moreover, studies have shown that for the S/P ratio, the trends are the most noticeable in spring^39^, at low to mid-elevations typically between 1500 and 2500 m near the climatological freezing level^32,37,39,40^. These elevations have been identified as the most vulnerable to warming^41^. The increase in melting level height would cause decrease in the S/P ratio^36^. Further, the positive trend of the Pacific-North American pattern contributes to increase in the freezing level height and decreases in the percent of precipitation falling as snow^42^. Therefore, the differences in the elevation and the freezing level height from station to station may be the reason why we have different trends in the SE/PE ratio even at the same latitude. More studies are encouraged in the future on the regional differences and the impact of large-scale climate variabilities on the SE/PE ratio.

A study that focused on western US argued that in regions where the S/P ratio is highly sensitive to temperature, the trends in the S/P ratio from 1916 to 2003 has already exceeded the historical variability^37^. Due to the limit of data length and data type, we cannot carry out analysis on a near-century long term variability of the SE/PE ratio. But considering that the changes in the S/P ratio are related to temperature and precipitation variability which are greatly influenced by human activities, we hope our work could encourage future studies on the physical mechanisms lying behind the noted SE/PE variability.

## Methods

### Data

The land station present weather reports are from two data sets: the National Centers for Environmental Prediction (NCEP) Automated Data Processing (ADP) Operational Global Surface Observations (ds464.0) from 1978 to February 2007^43^, and the NCEP ADP Global Surface Observational Weather Data (ds461.0) from October 1999 until present days^44^. The ds461.0 shares the same data source from the ds464.0, thus it can be used as an extension of the present weather observations after ds464.0 ended in 2007 (Supplementary Fig. S1).

The observation time at a weather station is not strictly fixed to the same hours among stations and grids, which would lead to jumps in the number of reports. To keep a roughly uniform observation counts among land stations, in our analysis we limited the present weather observations to four times a day at maximum by requiring the observation time to be within ± 1 h of the report times at 00, 06, 12, and 18 UTC. If more than one observation met the requirement, the report that was closest to the standard report hours (00, 06, 12, and 18 UTC) would be used.

After resampling, the ds464.0 and the ds461.0 data sets were merged to form a new data set that covers 1978–2019. Details about comparing and combining the two data sets can be found in Supplementary Fig. S1 and “Section 1: Merging data sets” in “Supplementary Information”.

For ocean data, we used the International Comprehensive Ocean–Atmosphere Data Set (ICOADS) release 3.0^45^. The ocean data were gridded into 5° (latitude) × 5° (longitude) boxes for further analysis. Because the observation times over ocean are random, the ocean reports were not resampled to keep the data volume. Data from 1978 to 2019 were used.

### Land station and ocean grid selection

We used the following criteria to select qualified land station and ocean grid for the data analysis. Between 1978 and 2019, the station must have at least 25 years and the grid must have at least 30 years where each year (1) has no less than 180 present weather observations out of 1460 (or 1464) maximum possible reports per year, and (2) has no less than 100 days with non-missing present weather reports. Strict data cleaning was then applied for each station and grid that meet the criteria. (See “Section 2: Data cleaning” in “Supplementary Information”). A total of 3915 out of more than 20,000 land manual stations and 713 ocean grids (5° × 5°) were analyzed. Most of them locate in the Northern Hemisphere (shown in Supplementary Fig. S2, “Supplementary Information”). Note that many stations have gaps in the period of records. When calculating trends, years that do not meet the requirements were excluded to avoid outliers (see the “Calculation of trends” section).

### Term definitions

The frequency of occurrence of rain (snow) is defined as the number of reports of rain (snow) divided by the number of total present weather reports within a certain time period expressed in fraction or percentage. It is also referred to as rain (snow) frequency. The snow event to precipitation event ratio is defined by the number of reports of snow divided by the number of total precipitation reports (that is, the sum of rain and snow reports). Referred to as SE/PE ratio, it depicts the likelihood for a precipitation event to be snow.

### Calculation of averaged rain (snow) frequency

The frequencies and the SE/PE ratio were calculated for stations or grids that met the selection standards introduced before. To derive the frequencies and the ratio, we counted the number of the 4-time-daily present weather reports for each identified land station, and for each 5° × 5° ocean grid box for each year. The overall annual frequencies and ratio for land or ocean were then calculated as the annual mean of all qualified stations or grids.

### Calculation of trends

Linear trend by method of least square fitting was calculated. Years with annual present weather reports less than 180 are excluded in the trend calculation. To calculate the trend for the rain/snow frequency from 1978 to 2019, there should be at least 25 years (for land) or 30 years (for ocean) where each year has at least 180 annual present weather reports out of 1460 (or 1464) maximum possible reports per year. Otherwise, no trend would be calculated and the station or grid would not be plotted in the figures. T-test is applied to examine the trends at 95% significance level.

### Notes on the “ww” codes

Only manual stations were examined in this study. A new coding practice was introduced by WMO around 1982, which slightly affected the number of reports^26,29^. Evaluations for 1986–2019 showed similar results as 1978–2019, suggesting that this change in coding practice does not influence the results. Missing ww reports were regarded as non-precipitation events.

## Supplementary Information

Supplementary Information.

## Data Availability

The NCEP ADP Operational Global Surface Observations (ds464.0) is available at https://rda.ucar.edu/datasets/ds464.0/. The NCEP ADP Global Surface Observational Weather Data (ds461.0) is available at https://rda.ucar.edu/datasets/ds461.0/. The International Comprehensive Ocean–Atmosphere Data Set (ICOADS) release 3.0 is available at https://rda.ucar.edu/datasets/ds548.0/. All map data are from Natural Earth and are publicly available at naturalearthdata.com. The Cartopy package (version 0.17.0) in Python was used to plot the maps (https://scitools.org.uk/cartopy/docs/v0.17/).
