# Author Correction: The latitudinal dependence in the trend of snow event to precipitation event ratio

**DOI:** 10.1038/s41598-021-99443-1

**Published:** 2021-10-05

**Authors:** Shangyong Shi, Guosheng Liu

**Affiliations:** Department of Earth, Ocean and Atmospheric Science, Florida State University, Tallahassee, FL USA

Correction to: *Scientific Reports* https://doi.org/10.1038/s41598-021-97451-9, published online 13 September 2021

The original version of this Article contained an error in Figure 2, where the ocean data in panel b was a duplication of this data in panel a. The original Figure 2 and accompanying legend appear below.

The original Article has been corrected.

**Figure 2:**
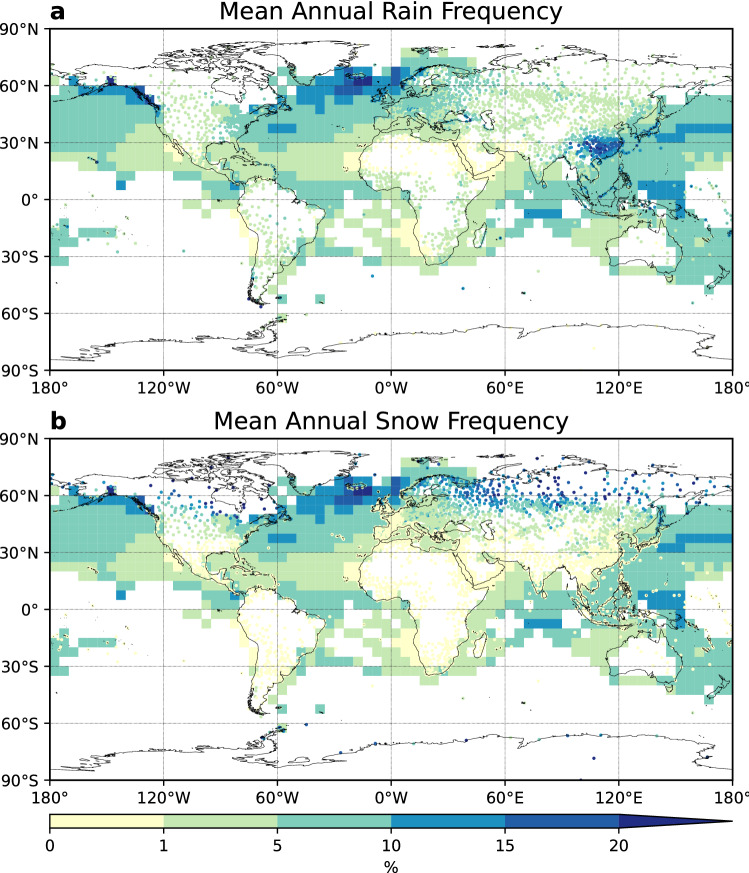
Mean annual rain and snow frequency of occurrences. (**a**) Mean annual rain frequency. (**b**) Mean annual snow frequency. Rain (snow) frequency of occurrence was calculated for each year, then the 42-year mean were computed for 1978–2019. This figure was plotted using Python 3.7.4 (https://www.python.org/downloads/release/python-374/).

