# Supplementary material for: The latitudinal dependence in the trend of snow event to precipitation event ratio
Source: Sci Rep. 2021 Sep 13;11:18112. doi: 10.1038/s41598-021-97451-9 (PMC8437944; doi:10.1038/s41598-021-97451-9)
Supplement: Supplementary file 1 — Supplementary Information. [file 41598_2021_97451_MOESM1_ESM.pdf]

Supplementary Information

The Latitudinal Dependence in the Trend of Snow Event to Precipitation Event Ratio

Shangyong Shi[1*] and Guosheng Liu[1]

[1]Department of Earth, Ocean and Atmospheric Science, Florida State University, Tallahassee, Florida, U.S.A.

Shangyong Shi: corresponding author, sshi2@fsu.edu

Guosheng Liu: gliu@fsu.edu

Section 1: Merging Data Sets

Both ds464.0 and ds461.0 were collected from the Global Telecommunications System (GTS) and were developed by NCEP. The report intervals in ds461.0 range from hourly to 3-hourly, thus, it is possible to match reports in ds461.0 with those 6-hourly and 3-hourly reports in ds464.0. Supplementary Figure S1 shows time series of four stations in ds464.0 (light color) and ds461.0 (dark color). The two data sets have the same number of reports and snow events during the overlapped period (2000-2006), which confirms that ds461.0 could serve as an extension of ds464.0.

After resampling two data sets to 4 times a day at maximum, we merged them to create a data set that covers 1978-2019. If the two datasets both have observations at the same time, we kept the one in ds464.0 (in most cases, these records are the same in the two data sets). Compared to directly using ds464.0 for 1978-2006, merging the data sets would make up some missing reports in ds464.0, thus improving the data quality.

Section 2: Data Cleaning

Between 1978 and 2019, the station must have at least 25 years and the grid must have at least 30 years where each year (i) has no less than 180 present weather observations out of 1460 (or 1464) maximum possible reports per year, and (ii) has no less than 100 days with non-missing present weather reports. Based on our selection standards, 4240 stations were picked out as candidates. Then we examined the data station by station. A station might not have continuous observations throughout the 42 years. Problems would occur when the number of ww reports suddenly drops to a very low value or recovers to the normal level of the station, or when the present weather observation becomes unavailable.

To avoid these problems, we cleaned the data as follows:

1) Removed the days when there are observations of other meteorological variables but no present weather.
2) Removed the years when the annual number of reports is less than 180 (this number corresponds to our selection standard).
3) Identified the years when the number of reports have sudden substantial changes and removed them. (Step 3 often comes together with Step 2.)
4) Identified stations with broken data. Among our candidate stations, those with observations less than 25 years were excluded from future analysis.

After cleaning, 3915 stations were left for calculations for means and trends (Supplementary Fig. S2a). Note that 220 stations have ww observations until around 2006 when the ds464.0 ended. The trends

calculated for these stations with data length between 25 and 29 years were marked as open circles in the figures.

The same data examination was done for the ocean grids. Since we selected relatively coarse resolution for ocean, the number of reports is adequate (mostly more than 1000 and never less than 200) and does not have sudden changes as the land stations do. Only around 10 grids have less than 30-year data. Selected ocean grids are shown in Supplementary Fig. S2b.

Section 3: Examples of Time Series.

The time series of number of ww reports, rain and snow events and the SE/PE ratio are presented to facilitate understanding the trends in SE/PE ratio. Supplementary Figure S3 shows an example of a tropical station. There are fewer snow events at this station, leading to a decreasing trend of the SE/PE ratio. Supplementary Figure S4 shows two examples. The station 24639 is having more snow and less rain events, and has increasing trend of the SE/PE ratio. The station 72773 has decreased number of both snow and rain events, and the relative change in snow is larger, leading to a decreasing trend in the SE/PE ratio.

Section 4: Correlation with Temperature

The correlation coefficient of annual mean temperature with annual mean SE/PE ratio for the 3915 land stations is shown in Supplementary Fig. S5.

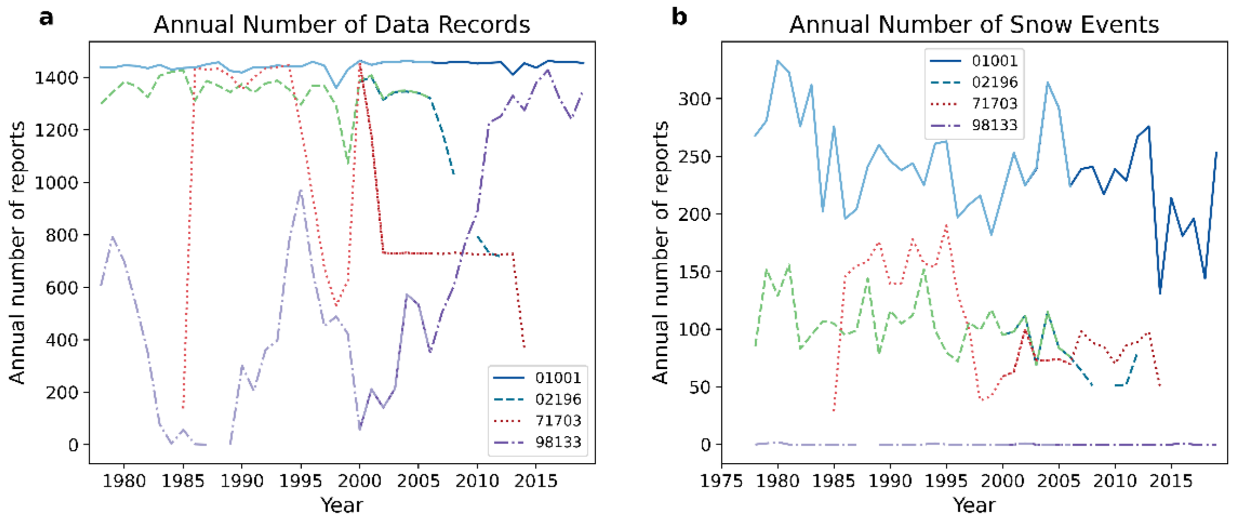

Supplementary Figure S1 **Comparison between ds464.0 and ds461.0**. **a.** Annual number of reports for four stations in ds464.0 (light color) and ds461.0 (dark color). **b.** Same as (**a**) but for the annual number of snow events. Special attention is given to the overlapped period of the two data sets (2000-2006).

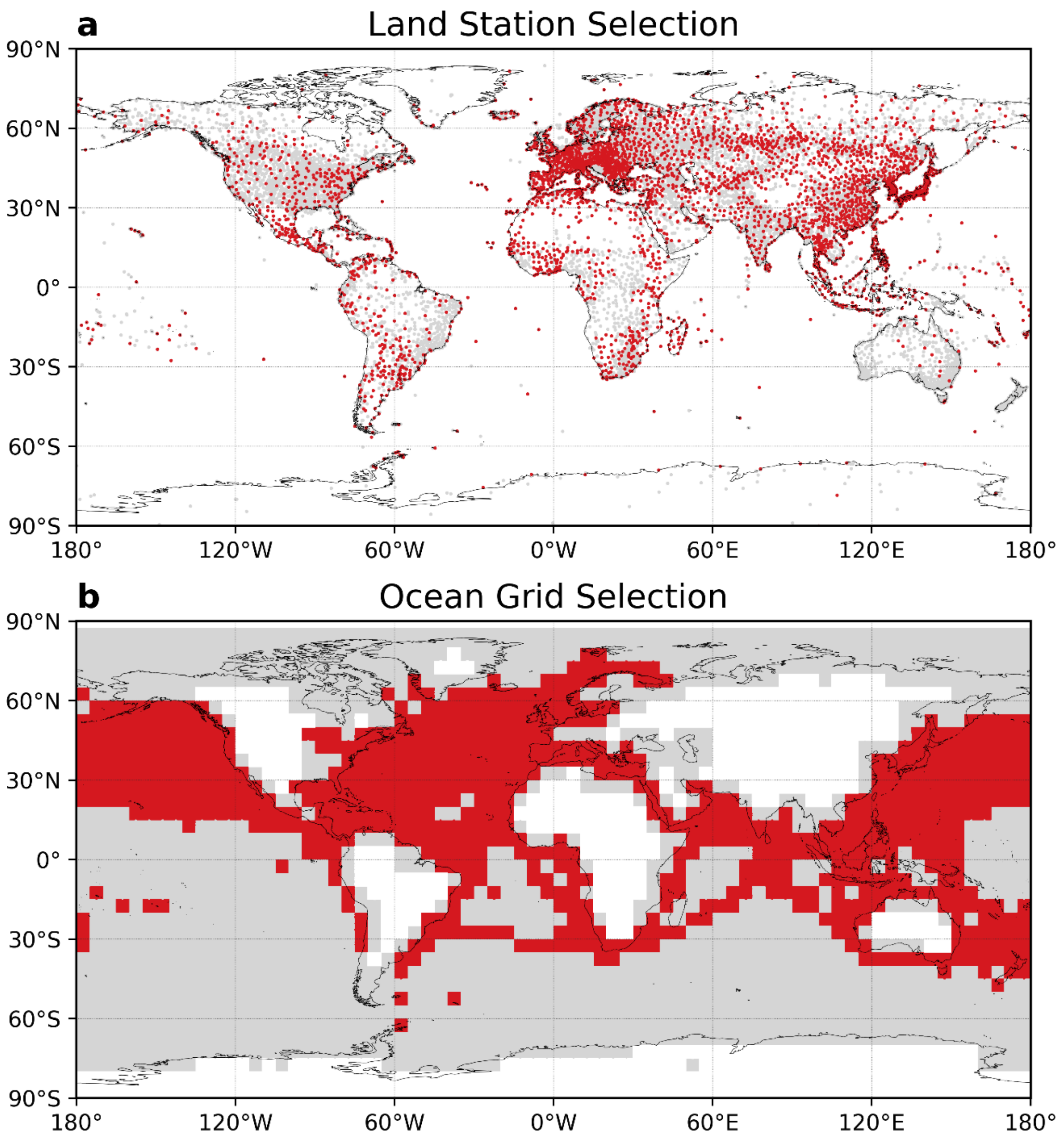

Supplementary Figure S2 **Data availability and selection for (a) land stations and (b) ocean grids in 5°×5°.** Gray means at least one observation during 1978 to 2019 is available at this station or grid, and red indicates qualified stations or grids following the selection standards. Between 1978 and 2019, the station or grid should have at least 25 or 30 years when (i) there are no less than 180 present weather reports out of 1460 (or 1464) maximum reports per year, and (ii) there are no less than 100 days with non-missing present weather reports. This figure was plotted using Python 3.7.4 (https://www.python.org/downloads/release/python-374/).

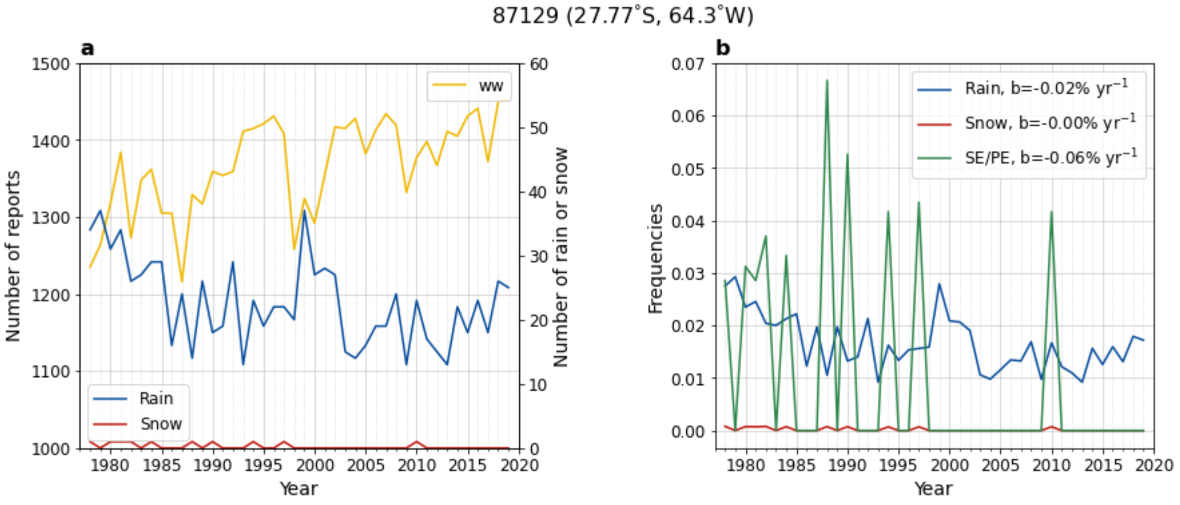

Supplementary Figure S3 **Time series of a tropical station (WMO ID: 87129) with a decreasing trend of SE/PE ratio**. **a,** annual number of ww events (yellow, left y-axis), rain events (blue, right y-axis) and snow events (red). **b,** The rain frequency (blue), snow frequency (red) and the snow event to precipitation event (SE/PE) ratio (green) for 1978-2019. The slope (b) of the linear regression line is shown in the legends with a unit of yr^{-1}.

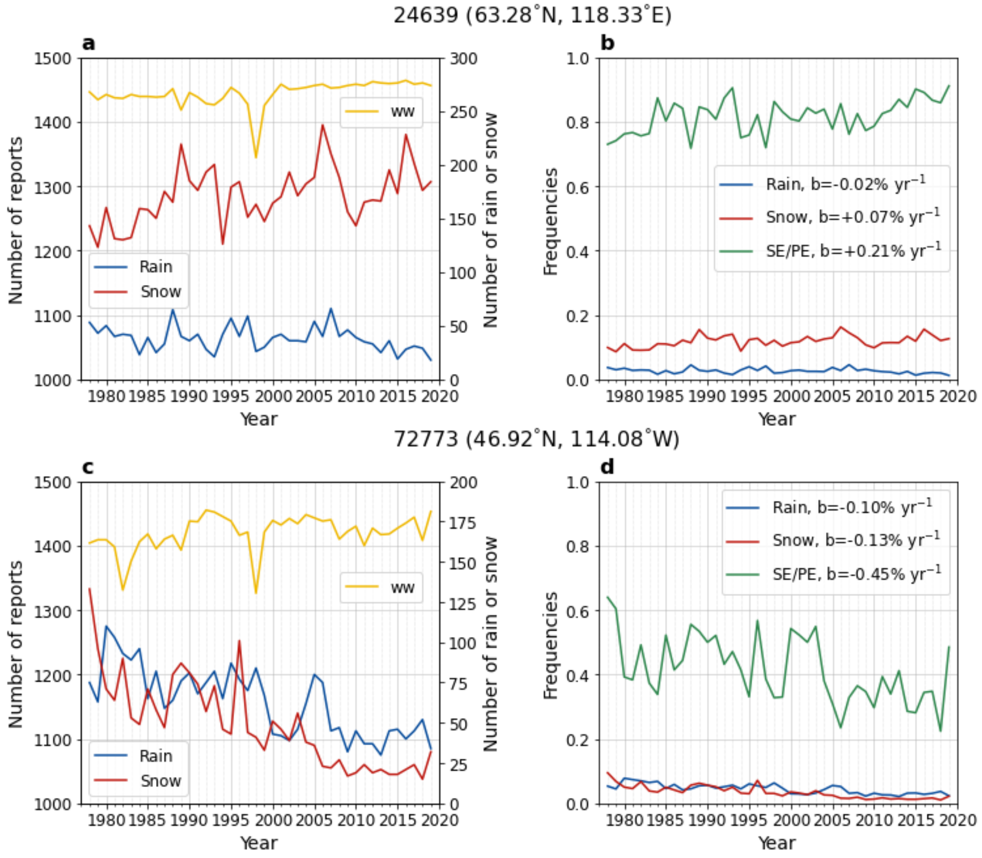

Supplementary Figure S4 **Time series of two stations outside the tropics.** Same as Supplementary Fig. S3, but for (**a, b**) land station 24639 (63.28°N, 118.33°E) and (**c**, **d**) land station 72773 (46.92°N, 114.08°W).

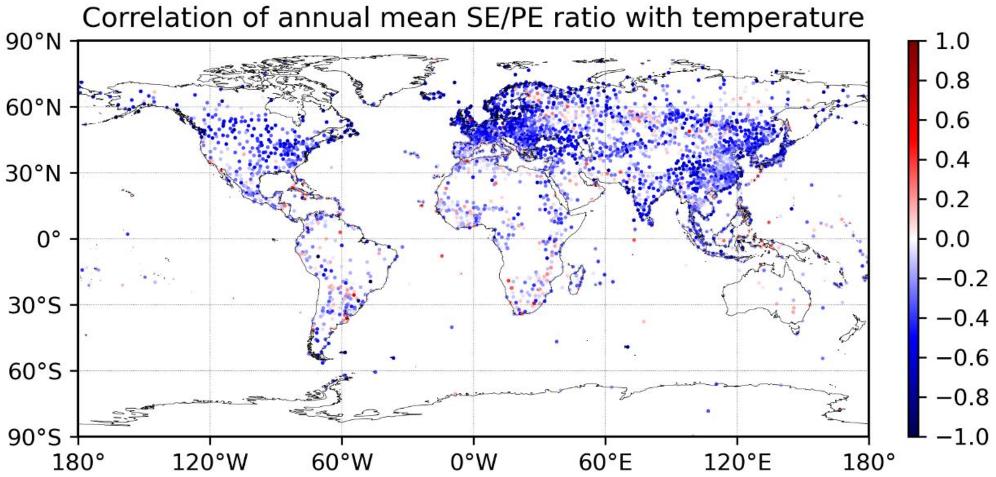

Supplementary Figure S5 **Correlation of the annual mean snow event to precipitation event ratio with annual mean temperature.** Spatial map of the correlation coefficient of the annual mean SE/PE ratio with annual mean temperature for the selected stations for 1978-2019. Only values on resampled hours in the combined data set were used to calculate the annual mean. This figure was plotted using Python 3.7.4 (https://www.python.org/downloads/release/python-374/).
